# Field resistance to wheat stem rust in durum wheat accessions deposited at the USDA National Small Grains Collection

**DOI:** 10.1002/csc2.20466

**Published:** 2021-05-30

**Authors:** Pablo D. Olivera, Worku D. Bulbula, Ayele Badebo, Harold E. Bockelman, Erena A. Edae, Yue Jin

**Affiliations:** ^1^ Dep. of Plant Pathology Univ. of Minnesota St. Paul Minnesota 55108 USA; ^2^ Ethiopian Institute of Agricultural Research Addis Ababa Ethiopia; ^3^ CIMMYT‐Ethiopia Addis Ababa Ethiopia; ^4^ USDA‐Agricultural Research Service Small Grains and Potato Germplasm Research Unit Aberdeen Idaho 83210 USA; ^5^ USDA‐Agricultural Research Service Cereal Disease Lab St. Paul Minnesota 55108 USA

## Abstract

Wheat stem rust, caused by *Puccinia graminis* f. sp. *tritici*, is a re‐emerging disease, posing a significant threat to durum wheat production worldwide. The limited number of stem rust resistance genes in modern cultivars compels us to identify and incorporate new effective genes in durum wheat breeding programs. We evaluated 8,245 spring durum wheat accessions deposited at the USDA National Small Grains Collection (NSGC) for resistance in field stem rust nurseries in Debre Zeit, Ethiopia and St. Paul, MN (USA). A higher level of disease development was observed at the Debre Zeit nursery compared with St. Paul, and the effective alleles of *Sr13* in this nursery did not display the level of resistance observed at the St. Paul nursery. Four hundred and ninety‐one (∽6%) accessions exhibited resistant to moderately susceptible responses after three field evaluations at Debre Zeit and two at St. Paul. Nearly 70% of these accessions originated from Ethiopia, Mexico, Egypt, and USA. Eight additional countries, namely Portugal, Turkey, Italy, Canada, Chile, Australia, Syria, and Tunisia contributed to 19% of the resistant to moderately susceptible entries. Among the 491 resistant to moderately susceptible accessions, 53.8% (n = 265) were landraces, and 28.4% (n = 139) and 11.4% (n = 55) were breeding lines and cultivars, respectively. Breeding lines and cultivars displayed a higher level and frequency of resistance than the landraces. We concluded that a large number of durum wheat accessions from diverse origins deposited at the NSGC can be exploited for diversifying and improving stem rust resistance in wheat.

## Core Ideas

- Identify new sources of stem rust resistance through field evaluations of durum wheat.
- 8,245 durum accessions deposited at NSGC evaluated in two field nurseries (Ethiopia and US).
- Presence of large number of entries from diverse origin that can be used for rust resistance.

**Abbreviations**
APR: adult plant resistance
COI: coefficient of infection
MR: moderately resistant
MS: moderately susceptible
NSGC: National Small Grains Collection
*Pgt*
: *Puccinia graminis* f. sp. *tritici*
R: resistant
Ssusceptible: susceptible
T: traces.

## 1 INTRODUCTION

Stem or black rust, caused by *Puccinia graminis* Pers.:Pers. f. sp. *tritici* Eriks. & E. Henn. (*Pgt*), is one of the most destructive diseases of durum wheat [*Triticum turgidum* L. sp. *durum* (Desf.) Huns.] and bread wheat (*T. aestivum* L.) worldwide. Severe devastations caused by stem rust epidemics were reported in the major wheat‐growing regions until the late 1950s (Roelfs, 1985; Saari & Prescott, 1985) when the disease was effectively controlled through the widespread use of host resistance and the eradication of the alternate host, common barberry (*Berberis vulgaris* L.) in Europe and the USA. Wheat stem rust is a re‐emerging disease, posing a significant threat to wheat production worldwide (Singh et al., 2015). The occurrence and spread of *Sr31*‐virulent races in the Ug99 race group in East Africa (Hale et al., 2013; Newcomb et al., 2016; Pretorius et al., 2000; Singh et al., 2015; Wanyera et al., 2006), coupled with other races causing severe epidemics and localized outbreaks in East Africa (Olivera et al., 2015), Europe (Bhattacharya, 2017; Lewis et al., 2018; Olivera Firpo et al., 2017), and Central Asia (Shamanin et al., 2020), indicates that the disease has reemerged as a major challenge to wheat production.

Tetraploid wheats have contributed genes for stem rust resistance, including *Sr7a*, *Sr8b*, several alleles of *Sr9*, *Sr11*, *Sr12*, alleles of *Sr13*, *Sr14*, *Sr17*, and *Sr8155‐B1* (McIntosh et al., 1995; Nirmala et al., 2017; Zhang et al., 2017). Many of these genes are widely used in common and durum wheat, contributing to the successful control of stem rust worldwide. For example, *Sr13* is a major component of stem rust resistance in durum wheat worldwide (Klindworth et al., 2007; Luig, 1983; Quamar et al., 2009; Singh et al., 2015), and it is the only gene in durum wheat that is effective against all variants in the Ug99 race group (Simons et al., 2011). The occurrence of *Pgt* races (JRCQC and TTRTF) with combined virulence to *Sr13b* and *Sr9e* (Olivera et al., 2012b; Olivera et al., 2019), has increased the vulnerability of durum wheat. The severe stem rust epidemic on durum wheat in Sicily (Italy) in 2016 caused by race TTRTF (Bhattacharya, 2017; Patpour et al., 2018) was indicative of the threat of stem rust to durum wheat production. Virulent *Pgt* races, including TTRTF, were recently detected in North Africa and the Middle East, both regions where durum wheat is an important crop (Hovmøller et al., 2020; Patpour et al., 2020). The limited availability of resistance to stem rust in durum wheat, coupled with the rapid occurrence and spread of virulent *Pgt* races, requires the identification and deployment of new and diverse resistance genes. Genetic resources, such as those maintained in germplasm banks, offer diverse sources to expand the genetic base of stem rust resistance in durum wheat. More than 78,000 accessions of durum wheat and its close relatives (other *T. turgidum* spp.) are deposited in germplasm collections around the world (Skovmand et al., 2005). The United States Department of Agriculture (USDA) National Small Grain Collection (NSGC) in Aberdeen (Idaho) maintains about 8,300 durum wheat accessions from more than 80 countries, which include landraces, obsolete cultivars, and modern breeding lines and cultivars. Breeding lines and cultivars represent the most immediate useful germplasm for breeding for disease resistance, but their contribution of new resistance genes may be limited because of the narrow genetic background of the pool of elite durum cultivars (Maccaferri et al., 2005; Singh et al., 1992). Durum landraces provide a useful source of genetic variability. These genetically diverse and locally well‐adapted materials derived from farmers’ selections provide a good source of genetic variability for crop improvement (Villa et al., 2006). However, as landraces are unimproved materials, more intensive pre‐breeding manipulations are required to transfer desired genes from them into advanced breeding lines (Skovmand & Rajaram, 1990). The objective of this work was to identify new sources of stem rust resistance through field evaluations of durum wheat accessions deposited at the NSGC germplasm bank.

**Core Ideas**

- Identify new sources of stem rust resistance through field evaluations of durum wheat.
- 8,245 durum accessions deposited at NSGC evaluated in two field nurseries (Ethiopia and US).
- Presence of large number of entries from diverse origin that can be used for rust resistance.

## 2 MATERIALS AND METHODS

### 2.1 Germplasm

A total of 8,245 spring durum wheat accessions deposited at the USDA NSGC (Aberdeen, ID, USA) were evaluated for 9 yr. The collection included 574 cultivars, 1,195 breeding materials, 5,688 landraces, and 788 accessions of uncertain improvement status from 82 countries. Among the countries, 17 contributed more than 100 accessions each, 32 contributed between 10 and 99 accessions, and 33 contributed fewer than 10 accessions (Supplementary Table S1).

### 2.2 Field stem rust evaluation

Field stem rust evaluations were conducted between 2009 and 2017 at the international durum stem rust nursery at Debre Zeit Agricultural Research Center in Ethiopia and at the University of Minnesota in St. Paul, MN (USA). At the Debre Zeit nursery, stem rust evaluations were performed in two seasons per year: the main (meher) season under rainfed conditions (June – November) and the off‐season under irrigated conditions (January – June). The Debre Zeit nursery was artificially inoculated with a local source of inoculum that consisted of races TTKSK (a variant in the Ug99 race group), JRCQC (combined virulence on *Sr13b* and *Sr9e*), and race TRTTF (appeared to be virulent to *Sr9e* and *Sr13a*) (Olivera et al., 2012b). Since 2014, race TKTTF, which was responsible for the severe stem rust epidemic in Ethiopia in 2013 (Olivera et al., 2015), was included as part of the field inoculum. In St. Paul, the nursery was artificially inoculated with a composite of six US races representing a diversity of virulence to specific resistance genes: TPMKC, RKRQC, RCRSC, QTHJC, QFCSC, and MCCFC. The virulence/avirulence profile of all the isolates used in this study is presented in Table 1. At the Debre Zeit nursery, entries were planted in double 1‐m row plots, whereas in St. Paul nursery, entries were planted as single 1‐m rows. Wheat cultivar ‘Red Bobs’ (cultivar 6255) or line LMPG‐6 was included at an interval of 50 lines as a susceptibility check. Continuous rows of stem rust spreader (i.e., mixture of susceptible lines) were planted perpendicular to all entries to facilitate inoculum buildup and uniform infection. In addition, the 20 stem rust differentials and lines carrying other relevant stem rust resistance genes were planted every season to monitor pathogen virulence in the nursery. Wheat lines and cultivars carrying *Sr13a* (ST464, Combination VII, and Khapstein/9*LMPG‐6) and *Sr13b* (Leeds and Sceptre) were also included to assess the effect of this resistance gene under field conditions in both nurseries. For details about the management of the nurseries and inoculation procedures at St. Paul and Debre Zeit, refer to Olivera et al., 2012a, 2012b. Disease assessment was done at the soft‐dough stage of plant growth. Because of differences in maturity among durum entries, three to four data points were recorded at weekly intervals, starting when the first entries reached the soft‐dough stage. Plants were evaluated for their response to infection (i.e., pustule type and size) (Roelfs et al., 1992) and terminal disease severity following the modified Cobb scale (Peterson et al., 1948). Infection response categories were: resistant (R), moderately resistant (MR), moderately susceptible (MS), and susceptible (S). Combinations of categories were recorded when two or more infection responses were observed on a single stem. Infection response categories R, R‐MR, and MR‐R were considered resistant, infection responses MR and MR‐MS were considered moderately resistant, and infection responses MS‐MR and MS with 30% or lower stem rust severity were considered moderately susceptible.

**TABLE 1:** Isolate designation, origin, and virulence phenotype of *Puccinia graminis* f. sp. *tritici* races used to evaluate resistance in durum wheat (*Triticum turgidum* sp. *durum*)

Race	Isolate	Origin	Avirulence	Virulence
TTKSK^a^	P14ETH02‐1	Ethiopia	*Sr24 36 Tmp*	*Sr5 6 7b 8a 9a 9b 9d 9e 9g 10 11 17 21 30 31 38 McN*
TRTTF	33Wonchi‐1	Ethiopia	*Sr8a 24 31*	*Sr5 6 7b 9a 9b 9d 9e 9g 10 11 17 21 30 36 38 McN Tmp*
JRCQC	PETH01DZ‐2	Ethiopia	*Sr5 7b 8a 9b 10 24 30 31 36 38 Tmp*	*Sr6 9a 9d 9e 9g 11 17 21 McN*
TKTTF	Digalu 1/1 Assasa	Ethiopia	*Sr11 24 31*	*Sr5 6 7b 8a 9a 9b 9d 9e 9g 10 17 21 30 36 38 McN Tmp*
TPMKC	74MN1409	USA	*Sr6 9a 9b 24 30 31 38*	*Sr5 7b 8a 9d 9e 9g 10 11 17 21 36 McN Tmp*
RKRQC	99KS76A‐1	USA	*Sr9e 10 11 24 30 31 38 Tmp*	*Sr5 6 7b 8a 9a 9b 9d 9g 17 21 36 McN*
RCRSC	77ND82A	USA	*Sr6 8a 9e 11 24 30 31 38 Tmp*	*Sr5 7b 9a 9b 9d 9g 10 17 21 36 McN*
QTHJC	75ND717C	USA	*Sr7b 9a 9e 24 30 31 36 38 Tmp*	*Sr5 6 8a 9b 9d 9g 10 11 17 McN*
QFCSC	06ND76C	USA	*Sr6 7b 9b 9e 11 24 30 31 36 38 Tmp*	*Sr5 8a 9a 9d 9g 10 17 21 McN*
MCCFC	59KS19	USA	*Sr6 8a 9a 9b 9d 9e 11 21 24 30 31 36 38*	*Sr5 7b 9g 10 17 McN Tmp*

^a^ Race nomenclature based on Roelfs & Martens (1988) and Jin et al. (2008).

In each evaluation year, 1,000 durum accessions from NSGC and checks were evaluated for field resistance in the main‐season nursery at Debre Zeit. Entries rated as resistant, moderately resistant, and moderately susceptible, with a maximum of 30% terminal disease severity (30 MS) in the Debre Zeit field nursery, were selected and further evaluated in the next off‐season nursery at Debre Zeit and the St. Paul nursery. Entries that remained resistant to moderately susceptible after the three field tests (two in Debre Zeit and one in St. Paul) were evaluated for one additional season in both nurseries. Mean disease severity and median infection response in both the Debre Zeit and St. Paul nursery evaluations were calculated for all the accessions that were resistant to moderately susceptible in all field tests. The median infection response for each accession was converted into a constant value and multiplied by the terminal disease severity to derive a coefficient of infection (COI; Stubbs et al., 1986). One‐way ANOVA and Duncan's Multiple Range Test were used to test the significance of the difference in the mean disease severity and the COI.

## 3 RESULTS

Uniform disease development across the field was observed in the Debre Zeit and St. Paul stem rust nurseries in all the seasons. Disease development was generally higher in the Debre Zeit nursery than the St. Paul nursery. In Debre Zeit, disease severity and infection response on the susceptible checks ranged between 50 and 90 S, with a mean value of 75 S, whereas in St. Paul, disease severity and infection response ranged between 50 and 80 S, with a mean value of 65 S. Infection observed on the differentials and lines carrying additional genes confirmed the virulence composition of the used inoculum in both nurseries. Unusual virulences were not detected in all the field seasons in Debre Zeit and St. Paul nurseries (data not shown). At the St. Paul nursery, lines carrying *Sr13a* (ST464, Combination VII, and Khapstein/9*LMPG) exhibited a moderately resistant to moderately susceptible response, whereas the two cultivars carrying *Sr13b* (Leeds and Sceptre) were highly resistant (0 to 10 R) (Table 2). At the Debre Zeit nursery, these five lines and cultivars, carrying *Sr13a* and *Sr13b*, exhibited high infection response and disease severity that ranged from 30 MS to 60 S (Table 2).

**TABLE 2:** Range of stem rust infection response and disease severity of durum wheat lines and cultivars carrying alleles of *Sr13* gene in field evaluations in Debre Zeit, Ethiopia and St. Paul, MN stem rust nurseries

		Debre Zeit nursery				St. Paul nursery			
Line/Cultivar	*Sr13* allele	Range of field reaction	Mean Severity	Median infection response	Mean COI^a^	Range of field reaction	Mean Severity	Median infection response	Mean COI
ST464	*Sr13a* (R1)^b^	30 S‐MS – 50 S^c^	43.0	S‐MS	40.0	40 MR – 50 MR‐MS	37.0	MR‐MS	17.9
Combination VII	*Sr13a* (R3)	30 MS‐S – 50 S	40.0	S‐MS	34.9	30 MR‐MS – 40 MS‐MR	48.2	MR‐MS	23.9
Khapstein/9*LMPG	*Sr13a* (R3)	30 MS – 60 S	45.0	MS‐S	39.0	40 MR‐MS – 60 MS	50.0	MS‐MR	31.6
Leeds	*Sr13b* (R2)	30 MS‐S – 60 S	44.3	S‐MS	42.0	0 – 5 R	3.1	R	0.3
Sceptre	*Sr13b* (R2)	30 S – 50 S	45.0	S	42.8	T R – 10 R	5.9	R	0.6

^a^ COI: coefficient of infection.

^b^ Based on Zhang et al. (2017).

^c^ Stem rust severity following the modified Cobb scale (Peterson, Campbell, & Hannah, 1948) and pustule type and size according to Roelfs, Singh, & Saari (1992). R, resistant; R‐MR, resistant to moderately resistant; MR‐R, moderately resistant to resistant; MR, moderately resistant; MR‐MS, moderately resistant to moderately susceptible; MS‐MR, moderately susceptible to moderately resistant; MS, moderately susceptible; S, susceptible.

**FIGURE 1:**
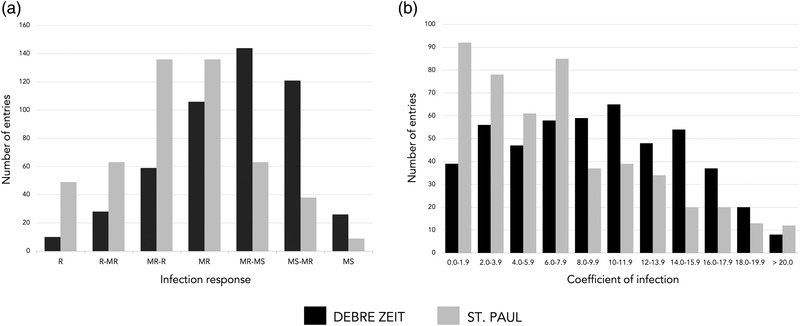
Number of entries in each stem rust response (A) and coefficient of infection (COI) (B) categories for 491 durum wheat (*Triticum turgidum* sp. *durum*) accessions exhibiting a resistant to moderately susceptible response in three field evaluations in Debre Zeit nursery (black) and two evaluations in St. Paul nursery (grey)

From the 8,245 spring durum wheat accessions evaluated in the main season at Debre Zeit, 1,787 (21.7%) exhibited a resistant to moderately susceptible response. These accessions were evaluated again at the Debre Zeit off‐season and St. Paul nurseries, and 657 (8.0% of total accessions) remained resistant to moderately susceptible after all three field tests. An additional evaluation of these accessions in both locations resulted in 491 accessions (6.0%) exhibiting a consistent reaction from resistant to moderately susceptible in the five field tests. Results of infection response and disease severity in each field evaluation, and mean severity, median infection response, and COI of the 491 accessions are presented in Supplementary Table S2. The mean coefficients of infection at Debre Zeit and St. Paul nurseries were 9.46 and 7.23, respectively (*p *< .001). The distribution of the 491 durum accessions according to infection response categories and COI in both nurseries showed a higher level of resistance in St. Paul than Debre Zeit (Figure 1).

**FIGURE 2:**
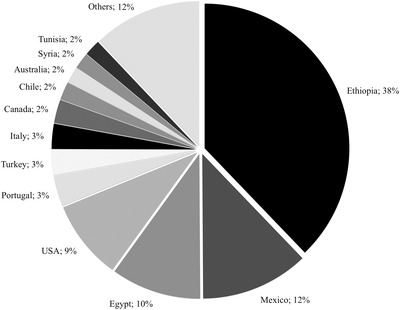
Origin and percentage distribution of the 491 durum wheat (*Triticum turgidum* sp. *durum*) accessions rated as resistant to moderately susceptible after five field evaluations in Debre Zeit (Ethiopia) and St. Paul, MN (USA)

Resistant to moderately susceptible accessions were identified from 37 countries, and nearly 70% of these accessions originated from four countries: Ethiopia (38%), Mexico (12%), Egypt (10%), and USA (9%) (Figure 2). Eight additional countries, Portugal, Turkey, Italy, Canada, Chile, Australia, Syria, and Tunisia, contributed to 19% of the resistant to moderately susceptible accessions (Figure 2). The majority of resistant accessions from Ethiopia (98%), Egypt (68%), Portugal (82%), and Turkey (86%) were landraces (Table 3). All the resistant accessions from Mexico, USA, and Canada were improved materials. Breeding lines and cultivars also constituted the majority of the resistant to moderately susceptible accessions from Chile, Australia, Italy, Syria, and Tunisia (Table 3). The frequencies of resistance differed between the countries of origin. Of the countries from where we tested more than 50 accessions, Egypt (n = 183) and Mexico (n = 237) exhibited a frequency of resistance of >20%, whereas accessions from Ethiopia (n = 1,529), Canada (n = 97), Chile (n = 57), Australia (n = 52), and Syria (n = 52) had a frequency of resistance between 10 and 20% (Table 3).

**TABLE 3:** Total number of durum wheat (*Triticum turgidum* sp. *durum*) accessions evaluated for stem rust resistance, and number and percentage of resistant to moderately susceptible breeding materials, cultivars, landraces, and entries of unknown origin according to country of origin

				Breeding material^a^		Cultivar		Landrace		Uncertain	
Country	Total number of entries	Number of resistant entries	% of resistant to moderately susceptible entries	Total	Resistant to moderately susceptible	Total	Resistant to moderately susceptible	Total	Resistant to moderately susceptible	Total	Resistant to moderately susceptible
Ethiopia	1,529	187	12.2	1	0	8	4	1,511	183	9	0
Mexico	237	60	25.3	189	50	34	7	5	0	9	3
Egypt	183	50	27.3	7	1	15	6	138	34	23	9
United States	557	44	7.9	484	37	63	7	0	0	10	0
Portugal	430	17	4.0	15	2	4	0	310	14	101	1
Turkey	1,188	14	1.2	5	0	12	2	1,117	12	54	0
Italy	302	14	4.6	105	4	107	7	62	2	28	1
Canada	97	12	12.4	61	9	35	3	0	0	1	0
Chile	57	10	17.5	32	5	9	2	4	1	12	2
Tunisia	310	8	2.6	21	5	24	2	250	1	15	0
Australia	52	8	15.4	28	3	14	2	2	0	8	3
Syria	52	8	15.4	22	7	0	0	30	1	0	0
France	93	7	7.5	34	2	28	4	4	0	27	1
Morocco	197	4	2.0	6	0	7	0	111	4	73	0
India	101	4	4.0	24	0	0	2	61	1	16	1
Argentina	26	4	15.4	8	1	8	1	3	0	7	2
Yemen	20	4	20.0	0	0	0	0	10	0	10	4
Algeria	240	3	1.3	7	2	16	0	206	1	11	0
Spain	148	3	2.0	9	0	8	1	107	1	24	1
Greece	92	3	3.3	7	0	6	1	75	2	4	0
Israel	38	3	7.9	8	1	18	2	8	0	4	0
South Africa	23	3	13.0	10	3	6	0	0	0	7	0
Kenya	8	3	37.5	4	2	2	0	0	0	2	1
Jordan	77	2	2.6	3	0	12	1	44	1	18	0
Serbia	22	2	9.1	0	0	0	0	18	1	4	1
United Kingdom	13	2	15.4	3	1	0	0	0	0	10	1
Brazil	2	2	100.0	2	2	0	0	0	0	0	0
Iran	929	1	0.1	0	0	0	0	929	1	0	0
Russian Federation	220	1	0.5	11	0	74	1	115	0	20	0
Afghanistan	68	1	1.5	0	0	0	0	64	1	4	0
Hungary	52	1	1.9	23	1	3	0	0	0	26	0
Saudi Arabia	21	1	4.8	0	0	0	0	7	0	14	1
Georgia	18	1	5.6	3	0	0	0	15	1	0	0
Oman	11	1	9.1	0	0	0	0	11	1	0	0
Croatia	10	1	10.0	0	0	0	0	9	1	1	0
Switzerland	9	1	11.1	1	1	3	0	0	0	5	0
Tajikistan	8	1	12.5	0	0	0	0	8	1	0	0
Others	805	0	0.0	62	0	58	0	454	0	231	0
TOTAL	8,245	491	6.0	1,195	139	574	55	5,688	265	788	32

^a^ Improvement status according to the USDA‐ARS National Small Grains Collection (Aberdeen, ID).

Based on the median infection response from the five field evaluations, 28.3% of the 491 accessions were classified as resistant (i.e., infection responses R, R‐MR, and MR‐R), 62.5% as moderately resistant (i.e., infection responses MR and MR‐MS), and 9.2% as moderately susceptible (i.e., infection responses MS‐MR and MS) (Table 4). The mean terminal disease severities were 12.6, 20.9, and 26.1% in resistant, moderately resistant, and moderately susceptible categories, respectively (Table 4). The mean COI were also significantly different between the respective categories (Table 4).

**TABLE 4:** Mean disease severity and coefficient of infection (COI) of 491 durum wheat (*Triticum turgidum* sp. *durum*) entries categorized as resistant, moderately resistant, and moderately susceptible in five field evaluations at the Debre Zeit and St. Paul stem rust field nurseries

Category	Mean disease severity	Mean COI	Number (%) of accessions
Resistant	12.6 c^a^	3.5 c	139 (28.3%)
Moderately resistant	20.9 b	9.5 b	307 (62.5%)
Moderately susceptible	26.1 a	15.9 a	45 (9.2%)
TOTAL	19.1	8.4	491 (100%)

^a^ ‘a’ ‘b’ and ‘c’ indicate statistically significant differences among resistant categories (*P *< .05).

Of the 491 selected accessions, 53.8% (n = 265) were landraces, and 28.4% (n = 139) and 11.4% (n = 55) were breeding lines and cultivars, respectively (Table 5). Only 4.7% of the landraces evaluated (n = 5,688) were classified as resistant to moderately susceptible, whereas 11.7 and 9.8% of the total evaluated breeding lines and cultivars, respectively, were classified into these resistant categories (Table 5). In addition to having a higher proportion of moderately resistant accessions, the mean disease severity and COI were significantly higher for the landraces (21.4 and 9.6, respectively) compared with the breeding lines (15.4 and 6.4) and cultivars (17.0 and 7.3) (Table 6). Twenty‐five accessions that exhibited the lowest COI are listed in Table 7. The elite resistant group of accessions consistently exhibited moderately resistant to resistant responses and low disease severity throughout five seasons of field evaluation. The group consisted of breeding lines, cultivars, and landraces from different countries.

**TABLE 5:** Improvement status of durum wheat (*Triticum turgidum* sp. *durum*) accessions classified as resistant to moderately susceptible to wheat stem rust in five field evaluations at the Debre Zeit and St. Paul stem rust nurseries

Improvement ​status^a^	Resistant to moderately susceptible accessions	Total number of accessions evaluated
Breeding material	139 (11.7%)	1,195
Cultivar	55 (9.8%)	574
Landrace	265 (4.7%)	5,688
Uncertain	32 (4.1%)	788
TOTAL	491	8,245

^a^ According to the USDA‐ARS National Small Grains Collection (Aberdeen, ID).

**TABLE 6:** Percentage of durum wheat (*Triticum turgidum* sp. *durum*) accessions belonging to each resistant category, and mean disease severity and coefficient of infection (COI) according to improvement status

Category	Breeding materials^a^	Cultivars	Landraces	Unknown
Resistant	41.6	38.2	19.8	39.4
Moderately resistant	51.4	49.1	71.1	54.6
Moderately susceptible	7.0	12.7	9.1	6.0
Mean disease severity	15.4 b^b^	17.0 b	21.4 a	18.8 b
Mean COI	6.4 b^c^	7.3 b	9.6 a	7.9 b

^a^ Improvement status according to the USDA‐ARS National Small Grains Collection (Aberdeen, ID).

^b^ ‘a’ and ‘b’ indicate statistically significant differences for mean disease severity among improvement status groups (*P *< .05).

^c^ ‘a’ and ‘b’ indicate statistically significant differences for mean COI among improvement status groups (*P *< .05).

**TABLE 7:** Stem rust infection response, disease severity, and coefficient of infection (COI) of the 25 most resistant durum wheat (*Triticum turgidum* sp. *durum*) accessions after five field evaluations in Debre Zeit and St. Paul stem rust nurseries

			Debre Zeit nursery			St. Paul nursery				
Entry	Country	Improvement status	1st Eval.	2nd Eval.	3rd Eval.	1st Eval.	2nd Eval	Mean severity	Median infection response	COI^a^
PI 428549	France	Cultivar	T R^b^	T R	5 R	T R	5 R	2.6	R	0.3
PI 519716	India	Uncertain	5 R	5 R	T R	T R	5 R	3.4	R	0.3
PI 520518	USA	Breeding line	5 R‐MR	5 R‐MR	5 R	T R	T R	3.4	R	0.3
PI 520348	Ethiopia	Cultivar	5 R	5 R	T R	5 R	5 R	4.2	R	0.4
CItr 15710	USA	Breeding line	5 R	5 R	5 R	5 R	5 R	5.0	R	0.5
PI 377886	Australia	Uncertain	5 R	5 R‐MR	5 R	5 R‐MR	5 R	5.0	R	0.5
PI 506469	USA	Cultivar	5 R‐MR	5 R	5 R‐MR	5 R	5 R	5.0	R	0.5
PI 519777	USA	Breeding line	0	T MR	T R	T R	10 R	2.6	R‐MR	0.5
PI 519380	Tunisia	Breeding line	T R	10 R	10 R‐MR	5 R	5 R	6.2	R	0.6
PI 435060	Croatia	Landrace	5 R‐MR	5 R	10 R‐MR	5 R	10 R	7.0	R	0.7
CItr 12920	Canada	Breeding line	5 R‐MR	10 R‐MR	T R	10 R	10 R	7.2	R	0.7
PI 520117	USA	Breeding line	5 R	10 R	10 R	5 R	5 R	7.5	R	0.8
PI 519445	USA	Breeding line	T R	T MR	5 R‐MR	5 R	10 R	4.4	R‐MR	0.8
PI 428454	Mexico	Breeding line	5 MR	5 MR	T R	5 R‐MR	5 R‐MR	4.2	MR‐R	1.3
PI 639888	USA	Breeding line	5 R‐MR	5 R	10 R‐MR	5 R	10 MR‐R	7.0	R‐MR	1.4
PI 519933	Tunisia	Breeding line	10 R‐MR	10 R‐MR	T R	10 MR	5 R	7.2	R‐MR	1.4
PI 367238	Italy	Cultivar	10 MR	20 MR	0	5 R	5 R	8.0	R‐MR	1.6
PI 278505	Spain	Uncertain	5 MR	5 R‐MR	5 MR	5 R‐MR	10 R‐MR	6.0	MR‐R	1.8
PI 470793	Ethiopia	Landrace	10 R‐MR	5 MR‐R	5 R‐MR	5 R	20 MR	9.0	R‐MR	1.8
PI 519469	Syria	Landrace	5 R‐MR	5 R	5 R‐MR	15 R‐MR	15 R‐MR	9.0	R‐MR	1.8
PI 639886	USA	Breeding line	10 R‐MR	5 R	10 MR	15 MR‐R	5 R‐MR	9.0	R‐MR	1.8
PI 520393	Tunisia	Breeding line	30 MR‐MS	T MR	5 R	5 R	5 R	9.2	R‐MR	1.8
CItr 15277	Italy	Uncertain	5 R‐MR	10 MR‐R	10 R	10 R	15 MR	10.0	R‐MR	2.0
CItr 15769	USA	Breeding line	15 MR‐MS	5 R	10 R‐MR	10 R	10 R	10.0	R‐MR	2.0
PI 324928	Argentina	Breeding line	15 MR	5 MR	5 R	20 R	5 R	10.0	R‐MR	2.0
Susceptible checks (Red Bobs / LMPG‐6)			70 S	80 S	70 S	70 S	65 S	71.0	S	71.0

^a^ COI (Stubbs, Prescott, Saari, & Dubin, 1986) calculated as the product of terminal disease severity and media infection response converted into a constant value. R = 0.1, R‐MR = 0.2, MR‐R = 0.3, MR = 0.4, MR‐MS = 0.5, MS‐MR = 0.6, MS = 0.7, MS‐S = 0.8, S‐MS = 0.9, S = 1.0.

^b^ Stem rust severity following the modified Cobb scale (Peterson, Campbell, & Hannah, 1948) and pustule type and size according to Roelfs, Singh, & Saari (1992). R, resistant; R‐MR, resistant to moderately resistant; MR‐R, moderately resistant to resistant; MR, moderately resistant; MR‐MS, moderately resistant to moderately susceptible; MS‐MR, moderately susceptible to moderately resistant; MS, moderately susceptible; S, susceptible; T, traces (less than 5% severity). .

## 4 DISCUSSION

Wheat stem rust is a re‐emerging disease, and recent epidemics and outbreaks indicate that it can pose a threat again to durum and bread wheat production. In particular, the occurrence of *Pgt* races with virulence to *Sr13b* and *Sr9e* (JRCQC and TTRTF) has increased the vulnerability of durum wheat to stem rust. The limited presence of effective stem rust resistance genes in improved materials underscore the need for identifying and incorporating new genes in durum breeding programs. To identify new sources of stem rust resistance in durum wheat, we evaluated 8,245 accessions of spring durum deposited at the NSGC for field resistance at two locations: the international durum stem rust nursery in Debre Zeit, Ethiopia and the US wheat stem rust nursery in St. Paul, MN. After five field evaluations at both locations, 491 (6%) accessions from 37 countries exhibited a resistant to moderately susceptible response. These 491 accessions were of diverse origins and can be exploited in wheat breeding programs for stem rust resistance.

To identify new effective stem rust resistance genes in durum wheat, it is crucial to evaluate the entries against *Pgt* races that are virulent to the most widely used resistance genes. The inoculum composition at the international stem rust durum nursery at Debre Zeit contains two important races with virulence to resistance genes common in durum: JRCQC with virulence against *Sr13b* and *Sr9e* (Olivera et al., 2012b) and TTKSK, which is virulent to *Sr8155B1* (Nirmala et al., 2017) and *Sr7a* (Jin et al., 2007). Olivera et al. (2012b) also reported an Ethiopian isolate, typed as race TRTTF, virulent on *Sr9e* and *Sr13a* at the seedling stage. Virulence on *Sr13a* by race TRTTF was not supported in the study done by Zhang et al. (2017). When TTKSK‐resistant durum wheats from North American breeding programs selected from field trials in Kenya in 2005 were evaluated at the Debre Zeit nursery, a large proportion became susceptible (Singh et al., 2015), suggesting that TTKSK resistance detected in Kenya, such as *Sr13* alleles common in durum, became ineffective to the race(s) in the Debre Zeit inoculum (Olivera et al., 2012b). High frequencies of resistant accessions have also been reported when durum germplasm was evaluated in field nurseries, where the inoculum composition lacks *Sr13* virulence (Meidaner et al., 2019; Pozniak et al., 2008; Singh et al., 2015). After nine years of field evaluations, our results indicated that, under field conditions in the Debre Zeit nursery, none of the alleles of *Sr13* conferred a sufficient level of resistance. The three alleles (haplotypes R1, R2, and R3) performed in a similar fashion, with a field reaction that ranged from 30 MS to 60 S. In the St. Paul nursery, *Sr13a* (R1 and R3) conferred a moderate level of resistance (MR‐MS to MS‐MR), which was comparable to the resistance to races in the Ug99 group in the Njoro, Kenya field nursery (Jin et al., 2007). Cultivars carrying *Sr13b* (Leeds and Sceptre) exhibited a much higher level of stem rust resistance (0 to 10 R). However, this high level of resistance may not be attributed to *Sr13b* alone, as these two durum wheat cultivars may carry additional resistance gene/s that are highly effective against the races used as inoculum at the St. Paul nursery. *Sr13* is a temperature‐sensitive gene that is more effective at temperatures ≥25°C (Zhang et al., 2017). However, the difference in the inoculum composition and overall disease pressure rather than temperature should explain the reduced effect of *Sr13* alleles at the Debre Zeit nursery, as temperature during disease development at Debre Zeit, in particular in the off‐season, was higher than at St. Paul. The lack of protection conferred by *Sr13* in the Debre Zeit nursery is also a strong indication of the need to broaden the base of stem rust resistance in durum wheat.

After the first evaluation at the Debre Zeit nursery, 21% of the entries exhibited resistant to moderately resistant infection responses. However, after completing five field evaluations in both locations, only 491 entries (6%) remained resistant to moderately susceptible. This difference is explained by 1) accessions resistant to Ethiopian *Pgt* races became susceptible when evaluated with US races, and 2) accessions that exhibited moderately resistant to moderately susceptible responses in the first evaluation in the Debre Zeit main season nursery, but, when exposed to an environment of higher disease pressure (off‐season nursery), they became moderately to fully susceptible. A higher temperature and availability of moisture through irrigation favored stem rust development in the off‐season nursery at Debre Zeit, resulting in a higher disease pressure. These results confirm the value of multiple field evaluations to adequately assess the level and stability of resistance in the evaluated germplasm. In a field evaluation at the Debre Zeit nursery in the main (rainfed) season, Chao et al. (2017) reported a higher frequency of resistant accessions at the Debre Zeit nursery in 429 durum accessions deposited at NSGC. As discussed by the authors, additional evaluations in the off‐season (irrigated) nursery could result in a reduction in the frequency of resistant accessions, as shown in this study. The similar environmental effect was evident when disease development at both locations was compared. We observed that disease severity and COI were consistently higher at the Debre Zeit nursery in the 491 accessions evaluated in all five seasons. At this nursery, there was a longer period of favorable weather conditions for disease development compared with St. Paul. The longer period of disease exposure and higher disease pressure resulted in an increased disease development at the Debre Zeit nursery.

The 491 resistant to moderately susceptible accessions can be divided into two main groups: cultivars and breeding lines mainly from North American (i.e., Mexico, USA, Canada) breeding programs and landraces from Ethiopia, Portugal, Egypt, and Turkey. About 10% of the cultivars and breeding lines evaluated in this study exhibited a resistant to moderately resistant response. Letta et al. (2013) reported 24% of 183 elite durum wheat cultivars and breeding lines from Italy, Morocco, Spain, Syria, Tunisia, southwestern USA, and Mexico to be resistant to moderately resistant when evaluated at the Debre Zeit nursery. This difference can be explained not only by the different origins of these elite materials but also because our study established a more stringent selection criteria that resulted in a lower percentage of resistant accessions.

The frequency and level of resistance exhibited by the breeding lines and cultivars were significantly higher compared with the landraces. Selection for stem rust resistance and the incorporation of effective resistance genes in modern breeding programs may have resulted in the higher resistance level in the breeding materials and cultivars. Most of the entries that exhibited the highest levels of resistance were breeding materials and cultivars. These advanced materials are good candidates for improving stem rust resistance in durum breeding programs. However, the contribution of new and diverse resistance genes from these improved materials may be limited because of the narrow genetic background of the pool of elite durum cultivars (Maccaferri et al., 2005; Quamar et al., 2009). On the contrary, landraces are a good source of genetic diversity and a potential source of new resistance genes. More than one third of the resistant accessions identified in this study were Ethiopian landraces, indicating their high potential as a source of stem rust resistance. Ethiopia is considered a secondary center of origin for tetraploid wheats (Kabbaj et al., 2017), and Ethiopian landraces have been regarded as a separate subspecies (sp. *abyssinicum*) of *T. turgidum* (Mengistu et al., 2015). Ethiopian durum landraces are morphologically distinct (Pecetti et al., 1992), and a high level of both phenotypic (Mengistu et al., 2015) and genotypic (Alemu et al., 2020) diversity has been reported. The level of resistance in Ethiopian durum landraces may be a result of thousands of years of co‐evolution with the stem rust pathogen in the central highlands of Ethiopia (Amogne et al., 2000) and an exposure to a diverse stem rust pathogen population (Admassu et al., 2009; Olivera et al., 2015). A broader and diverse basis of stem rust resistance may be needed to provide protection from the current pathogen races, such as JRCQC, that appear to have broader virulence against durum wheat (Hundie et al., 2019; Olivera et al., 2012b). Previous studies (Ataullah, 1963; Beteselassie et al., 2007; Bonman et al., 2007; Kenaschuk et al., 1959) have also identified Ethiopian landraces as a source of stem rust resistance genes. In particular, the Ethiopian landrace ST464 is the donor of *Sr13a*, an effective allele of *Sr13*, which is one of the most important stem rust resistance genes in durum wheat (Klindworth et al., 2007; Zhang et al., 2017). Seedling evaluations with multiple races to further characterize these resistant selections are in progress and will be useful to select landraces with unique resistance to broaden the stem rust resistance in wheat breeding programs. This study also identified resistant landraces from Egypt, Portugal, and Turkey. Durum landraces from these countries are also highly diverse (Akond & Watanabe, 2005; Altıntaş et al., 2008; Soriano et al., 2016), but only landraces of Egyptian origin exhibited a high frequency of stem rust resistance. Only a limited number of resistant accessions was observed from Turkey (i.e., 14 out of 1,188), which disagrees with the results reported by Bonman et al. (2007). Although accessions evaluated by Bonman et al. (2007) were also a part of the NSGC, that study was based only on resistance to US races.

Stem rust resistance in durum wheat relies on a limited number of major genes and adult plant resistance (APR) genes that have not been reported to be widely used in modern cultivars. Very few studies have described the presence of adult plant or slow rusting resistance in durum wheat (Hare, 1997; Hei et al., 2015; Toor et al., 2009), indicating the potential for identifying new APR resistance genes that will help improve durability of stem rust resistance. For example, *Sr2*, the most important APR gene in common wheat was transferred from Yaroslav emmer (McFadden, 1930), the tetraploid wheat progenitor of cultivated durum. To identify accessions with potential new APR genes, we are in the process of characterizing these resistant to moderately susceptible accessions using a wide range of *Pgt* races, including those used in both nurseries. Resistant accessions that exhibit susceptible reaction in all seedling evaluations can be investigated for the presence of APR. As the phenotypic effect of APR genes is relatively minor, it is expected that accessions carrying an APR gene would not exhibit a strong resistant response. For this reason, accessions consistently exhibiting a moderately susceptible response, with a relatively low disease severity (≤30% stem rust terminal disease severity), were included in our selection group for further studies on APR.

Seedling evaluations with multiple stem rust races, in combination with marker analysis, are needed to allow a relatively accurate postulation of the known major resistance genes deployed in durum wheat. Genotyping of the 491 resistant to moderately susceptible accessions with a 90K SNP platform is planned to identify and map new quantitative trait loci associated with stem rust resistance.

## 5 CONCLUSIONS

The results from this study demonstrated that durum wheat accessions deposited at the USDA National Small Grains Collection provided a good and diverse source of stem rust resistance. Four hundred and ninety‐one accessions were found to exhibit a resistant to moderately susceptible response after five field evaluations in Debre Zeit (Ethiopia) and St. Paul, Minnesota (USA). These accessions could be exploited for improvement of stem rust resistance in both durum and common wheat. A higher level and frequency of resistance was observed in cultivars and breeding lines compared with landraces. The landraces from different geographic origins have the potential to contribute a diverse source of new genes. Seedling evaluations and genotyping of these resistant germplasms will facilitate the characterization and mapping of effective stem rust resistance genes that can be incorporated into adapted backgrounds.

## AUTHOR CONTRIBUTIONS

Pablo D. Olivera: Conceptualization, Data curation, Formal analysis, Investigation, Methodology, Writing‐original draft, Writing‐review & editing; Worku D. Bulbula: Investigation, Writing‐review & editing; Ayele Badebo: Investigation, Writing‐review & editing; Harold E. Bockelman: Resources, Writing‐review & editing; Erena A. Edae: Formal analysis,Software,Visualization; Yue Jin: Conceptualization, Investigation, Methodology, Project administration, Supervision, Writing‐review & editing.

## CONFLICT OF INTEREST

The authors declare no conflict of interest.

## Supporting information

**Supplementary Table S1**: Origin and improvement status of durum wheat (*Triticum turgidum* sp. *durum*) accessions evaluated for stem rust field resistance.

**Supplementary Table S2**. Disease severity, infection response, and COI of 491 durum wheat (*Triticum turgidum* sp. *durum*) evaluated stem rust field nurseries at Debre Zeit (Ethoipia) and St. Paul, MN (USA)
